# Mental Nursing

**Published:** 1900-03

**Authors:** 


**Mental Nursing.** By WILLIAM HARDING, M.D. Pp. viii., 91. London: The Scientific Press, Limited. 1899.

During the past few years great advance has been made in the status of attendants on the insane, from the "keeper" of old to the intelligent nurse of the present, and for their still further improvement the new edition of *Mental Nursing* is specially adapted. The sections on Elementary Anatomy and Physiology and First Aid have been omitted, and replaced by others of more immediate value. The book now forms a sound guide for the treatment of the insane from the practical nursing point of view, and as such it can be strongly recommended. The author shows an intimate knowledge of the difficulties and onerous duties of nurses; their responsibilities are pointed out and encouragement is given to overcome the difficulties by an intelligent interest in, and study of, the various phases of mental disease.

